# Quantitative Evaluation of Growth Plates around the Knees of Adolescent Soccer Players by Diffusion-Weighted Magnetic Resonance Imaging

**DOI:** 10.1155/2015/482017

**Published:** 2015-11-26

**Authors:** Zmago Krajnc, Mitja Rupreht, Matej Drobnič

**Affiliations:** ^1^Department of Orthopaedic Surgery, University Medical Centre Maribor, Maribor, Slovenia; ^2^Department of Radiology, University Medical Centre Maribor, Maribor, Slovenia; ^3^Medical Faculty, University of Maribor, Maribor, Slovenia; ^4^Department of Orthopaedic Surgery, University Medical Centre Ljubljana, Ljubljana, Slovenia

## Abstract

*Purpose*. To quantitatively evaluate growth plates around the knees in adolescent soccer players utilizing the diffusion-weighted MR imaging (DWI).* Methods*. The knees and adjacent growth plates of eleven 14-year-old male soccer players were evaluated by MRI before (end of season's summer break) and after two months of intense soccer training. MRI evaluation was conducted in coronal plane by PD-FSE and DWI. All images were screened for any major pathological changes. Later, central growth plate surface area (CGPSA) was measured and the apparent diffusion coefficient (ADC) values were calculated in two most central coronal slices divided into four regions: distal femur medial (DFM), distal femur lateral (DFL), proximal tibia medial (PTM), and proximal tibia lateral (PTL).* Results*. No gross pathology was diagnosed on MRI. CGPSA was not significantly reduced: DFM 278 versus 272, DFL 265 versus 261, PTM 193 versus 192, and PTL 214 versus 210. ADC decrease was statistically significant only for PTM: DFM 1.27 versus 1.22, DFL 1.37 versus 1.34, PTM 1.13 versus 1.03 (*p* = 0.003), and PTL 1.28 versus 1.22.* Conclusions*. DWI measurements indicate increased cellularity in growth plates around knees in footballers most prominent in PTM after intense training. No detectable differences on a standard PD-FSE sequence were observed.

## 1. Introduction

Soccer is the most popular and widely played sport worldwide, especially among children and adolescents [1]. The players are notorious for their bow-legs and this general observation has recently been proven scientifically: studies by Chantraine and Witvrouw et al. confirmed an evident association between soccer playing and genu varum [2, 3]. Soccer playing, particularly at higher competition levels on a daily basis, induces an inordinate amount of load and torque in the involved extremity [2, 3]. In a growing skeleton, these forces are transferred further onto growth plates that are subsequently asymmetrically activated [4–6]. Varus deformity in boys typically arises between the ages of 13 to 15, when the skeletal growth spurt is reached [2, 3, 7, 8]. Varus causes medial knee compartment overloading and increases a risk for an early cartilage failure [9–12]. Bow-legs additionally predispose athletes to patellofemoral pain syndrome and meniscal lesions [8]. Current musculoskeletal MRI techniques may detect acute physeal injuries and their late consequences [13, 14], but they are not able to reveal chronic physeal dysfunction induced by the repetitive loading. Diffusion-weighted MR imaging (DWI) is a novel addition to the MR sequences which provides quantitative information on microscopic movements of water at the cellular level [15]. In musculoskeletal system, DWI has been particularly useful for the differentiation of metastatic from osteoporotic vertebral fractures, for the evaluation of bone marrow pathology, such as infection and haematological malignancies, and also for the detection of bone metastases and their response to treatment [15–20]. The aim of the current study was to evaluate usage of DWI in the chronic, sports activity-related disturbances of growth plates. To the best of our knowledge, this is the first time DWI was used for evaluation of the growth plates around the knees. We hypothesized that DWI-MRI is able to detect and quantify water diffusibility changes in growth plates around the knees of adolescent soccer players before and after intensive sports participation.

## 2. Materials and Methods

The study was designed as a 4-month single-centre case series. The protocol was approved by the National Medical Ethics Committee (number 86/02/13). The work was conducted in accordance with the Declaration of Helsinki (1964).

### 2.1. Participants and Sports Activity

The study group comprised eleven asymptomatic junior members U14 of the soccer club NK Maribor, Slovenia, who had a history of soccer training for a minimum of 3 years. Informed consent was obtained from all individual participants and their parents. The following exclusion criteria were set: history of lower limb surgery or any serious knee injury, musculoskeletal abnormalities or systemic disease with a possible impact on joints, or presence of metallic foreign bodies that would had prevented MRI examination. Players' general data and physical examination (height, weight, hip, and knee range of motion measurements) were acquired upon inclusion. Clinical measurements of lower limb mechanical alignment were conducted in a weight-bearing position with a calliper, similarly as previously described [7, 21]. Demographic and anthropometric data are summarized in Table 1.

The initial examinations and baseline MRIs were performed at the end of “summer soccer vacation,” which had lasted from mid-May 2014 till mid-June 2014. During this one-month period the players were not involved in any systematic soccer training. They were allowed to conduct only low intensity running, swimming, and cycling. No cutting and pivoting sports were performed during this period. The players were asked to fill in daily activities diary. After this quiet period, the players were enrolled into preparations for the new season. They followed an intense practice routine of circuit training workouts, aerobic and anaerobic exercises with and without the ball, high intensity running (sprinting and jogging), plyometric and isometric exercises, and soccer training skills (kicking, ball control, heading, dribbling, passing, and tackling). They were also involved in the preparation matches. Participants were active in the training process between 1.5 and 3.5 hours daily, 6 days a week. The second MRI was conducted after two months of such training, within 24 hours after their last sporting activity.

### 2.2. MRI Acquisition

**Figure 1:**
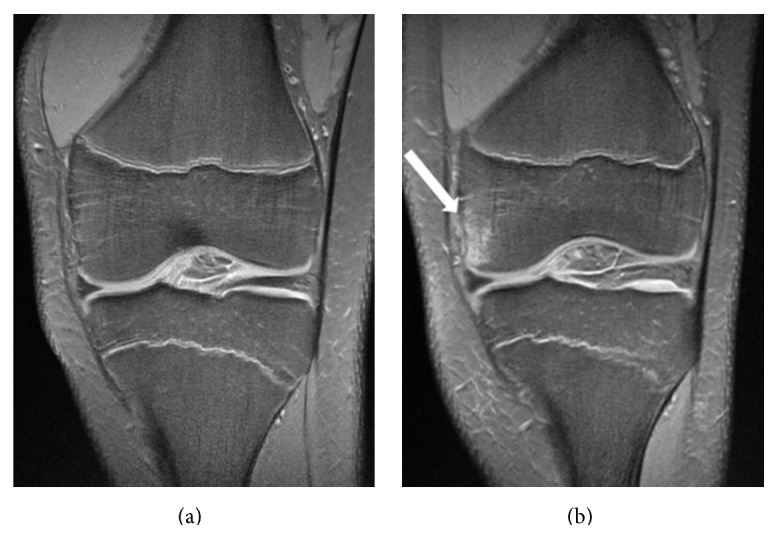
Coronal PD fat-saturated FSE images show left knee of a 14-year-old soccer player before (a) and two months after (b) seasonal training. Note the area of the increased signal intensity in the anterior aspect of the medial femoral condyle in (b) (arrow) compared to (a) representing bone marrow oedema reflecting stress reaction or contusion during the training.

**Figure 2:**
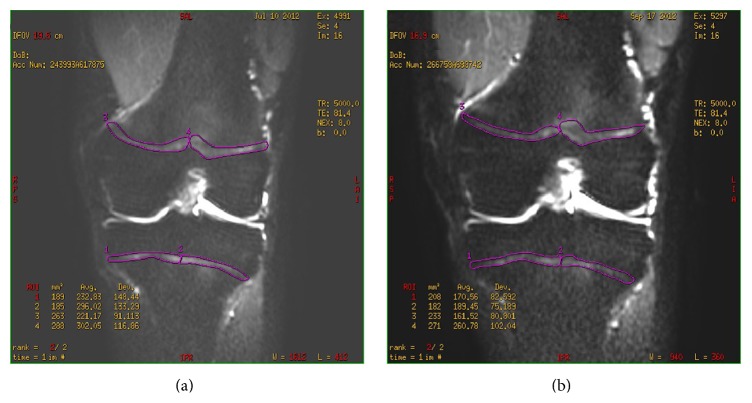
A sample of DWI analysis in a 14-year-old soccer player before (a) and two months after (b) sport activity. First, the coronal surfaces in the most central MRI were encircled (purple line). Each growth plate surface area was then halved to form four regions of interest for the analysis. An apparent diffusion coefficient (ADC) was calculated in each of these regions.

Both MRI scans were performed on a 3 T Signa Excite (General Electric, Waukesha, WI, USA) MRI scanner using eight-channel transmit-receive knee coil. Both knees were scanned in 7 players and only dominant leg was scanned in 4 players due to limited parent consent. MRI examinations were first performed in the proton-density fast spin-echo (PD-FSE) sequence with eighteen fat-suppressed slices in the coronal plane: TR (repetition time) = 2080 ms, TE (echo time) = 11 ms, ETL = 6, ST (slice thickness) = 3 mm, spacing = 0.3 mm, FOV (field of view) = 18 cm, and matrix = 384 × 384 (Figure 1). This pulse sequence has an excellent spatial and contrast resolution and it is one of the most commonly used in the routine knee MRI. It is clinically utilized for diagnosing the areas of bone marrow oedema (BME) as well as the analysis of the articular cartilage, menisci, and cruciate ligaments. DWI was performed with the echo-planar imaging (EPI) method. Ten 7 mm slices were acquired with 1 mm gap, using the spin-echo single shot technique at TR/TE = 8000/75 ms, 20 cm FOV, and 160 × 256 matrix. Two image acquisitions were performed for each DWI: one without (*b* = 0 s/mm^2^) and the other with diffusion weighting (*b* = 400 s/mm^2^) with the inferior-superior direction of gradient orientation. FOV included the distal femur and proximal tibia in a coronal plane thereby including the distal femoral and proximal tibial metaphysis (Figure 2).

### 2.3. MRI Analysis

All MRI analyses and measurements were performed by consensus of a musculoskeletal radiologist with 10 years of experience (MR) and an orthopaedic surgeon (ZK) being blind to the study group and the examination time. They were performed 2 months after the last MRI to minimize bias resulting from the consensus reading.

Coronal fat-suppressed PD-FSE images were first analysed for any gross pathology in and around the knee. Screening for BME was then performed. BME was defined as an area of visually detected clearly increased signal intensity in the bone marrow at least on two consecutive PD-FSE images, measuring between 0.5 cm² and 1.5 cm². Larger homogenous areas of slightly increased signal intensities, found predominantly in the diaphysis of femur and tibia, were not considered as BME, since they represent areas of active red marrow, which may be normally present in this age group [22].

For DWI analyses, the region of interest (ROI) encircled the border of the growth plates of femur and tibia in two consecutive most central coronal slices. At each slice, ROIs were divided into medial and lateral half (Figure 2) yielding four regions: distal femur medial (DFM), distal femur lateral (DFL), proximal tibia medial (PTM), and proximal tibia lateral (PTL). In the follow-up examination, the same slices were meticulously chosen for the analyses. From two DWI image sets of different *b* values, apparent diffusion coefficient (ADC) maps were calculated. This was followed by the calculation of ADC values (mm/s × 10^−3^) for each region with the subsequent average of both slices. Also, the area of each region was recorded and the average was calculated from both consecutive slices representing the central growth plate surface area (CGPSA) value (mm^2^).

### 2.4. Data Analysis

All the data are presented as mean values with SD. Preactivity to postactivity values of* CGPSA* and the ADC in each of the growth plate regions were compared by Student's *t*-test for paired samples with a statistical significance set to *P* < 0.05. The computer software IBM SPSS Statistics, Version 20, was used. Post hoc power analysis of ADC preactivity to postactivity values in the proximal tibia medial growth plate (PTM) showed an effect size of 0.80 and a statistical power of 0.95 (calculated with G^*∗*^Power 3.1.7, Universität Kiel, Germany).

## 3. Results

There was no gross pathology of cartilage, bone, ligaments, or menisci diagnosed on preactivity or on postactivity images. No intra-articular effusion was found in any case. Bone marrow oedema was detected in three knees of different individuals, always in the medial femoral condyle (Figure 1). In one knee, it was present before and after the soccer training, whereas in the other two it was diagnosed only at the follow-up examinations. CGPSA of all growth plates was not significantly reduced: DFM 278 versus 272; DFL 265 versus 261; PTM 193 versus 192; and PTL 214 versus 210. A decrease of ADC in all four growth plates occurred during the follow-up but it was statistically significant only for PTM: DFM 1.27 versus 1.22; DFL 1.37 versus 1.34; PTM 1.13 versus 1.03 (*p* = 0.003); and PTL 1.28 versus 1.22 (Table 2 and Figure 2).

## 4. Discussion

The aetiology of genu varum in soccer is thought to be multifactorial: ranging from natural selection of players with a genetic predisposition to varus (varus knees have some advantages for soccer performance) to mechanical overload of proximal medial tibial physis [7]. The susceptibility of growth plates to injury appears to be especially pronounced during the period of rapid pubescent growth spurt [13, 23]. An accumulating number of clinical reports indicate that intensive sport training may precipitate pathological changes of the growth plate and even produce growth disturbance [13]. An increased varus deviation around the knees of soccer players in comparison to the same aged peers was also observed during this time period [3, 7, 8, 10, 24]. Clinically measured axial deviations of lower extremities in our study group (mean ICD was 24 mm) were comparable to the reports of other authors (ICD from 22 to 33 mm) for the age matched soccer players [3, 7, 8]. Proximal tibial growth changes due to repeated stress over the open growth plates may be a possible mechanism of this axis deviation. Several factors other than running and cutting manoeuvres unique to the soccer may play a role as deforming forces of which the ball kicking deserves special attention for its additional torque movement which is unique to soccer when compared to other sports. However, the exact mechanism or activity that leads to increased medial growth plate stress remains enigmatic.

The histological evaluation of growth plates in humans is limited to the material retrieved at the surgical procedure, epiphysiodesis, which cannot be used for systematic studies of sporting population. Conventional radiography, CT, and MRI had been used in the assessment of growth plates injuries in the past [13, 25, 26]. Radiological diagnoses were based on the widening of the physis, irregularity of the metaphyseal line, and fragmentation or separation of the metaphysis [13, 14]. These radiological findings become visible only after a period of a pain interval when an athlete is brought to the radiological examination [13]. On the contrary, they fail to show any changes in asymptomatic adolescent athletes. To the best of our knowledge, DWI has not been utilized for the evaluation of the growth plates around the knees yet. It represents a novel diagnostic tool in which image contrast is related to the random motion of water protons, which differs in various tissue environments. It therefore noninvasively reflects the tissue organizational features, principally its cellularity [15]. Diffusion weighting in the spin-echo echo-planar T2 weighted sequence is achieved by two additional gradient pulses of equal magnitude and polarity symmetrically positioned relative to the refocusing RF pulse. The degree of diffusion weighting (*b* value) is determined by the amplitude of the gradient pulses, as well as by their duration and spacing. Two DWI acquisitions with different *b* values enable calculation of ADC map. Higher ADC values correspond to elevated diffusion in the extracellular space; the motion of water is less restricted [20]. In areas with high cellularity, molecular water mobility is impeded, yielding lower ADC values [20]. One of the most important findings of this study is the feasibility of DWI-MRI to detect subtle, activity-related changes in the growth plates around the knees of adolescent soccer players. The calculated ADC values in the growths plates were lower than mean values for free water (2.80 × 10^−3^ mm^2^/s at 37°C) [16], but higher than ADC values for normal bone marrow (0.15 to 0.23 × 10^−3^ mm^2^/s) [16, 17]. Decreasing ADC values at the follow-up examinations of adolescent soccer players, although not statistically significant, may reflect an increased cellularity in the growth plates, possibly owing to the maturation. Significantly lower ADC values in PTM growth plate disclose additional reduction of water mobility in the extracellular space in this region. This suggests that cellular structures in this zone are even higher packed as a response to the extreme repetitive rotational and pressure forces on the physes. This is consistent with histological findings in animal studies (more numerous chondrocytes, a notable increase in the hypertrophic cell zone, and a progressive disorganization of the layers and chondrocyte columns) after their growth plates were exposed to a compressive external impact [27, 28]. Consequently, we can presume that higher cellularity in medial tibial growth plate indicates the greatest impact of soccer training on the medial tibial growth plate. This is in accordance with the higher incidence of varus angulation among soccer players at the end of growth spurt [3, 7, 8]. It is also in line with general varus deformity of the knee, which is typically caused by deficiency in the medial tibia plateau, since distal femoral surface usually remains in valgus to neutral alignment to the long axis of femur [29]. Preserved CGPSA during the follow-up could be probably related to a relatively short follow-up period. This finding further emphasizes the importance of DWI for detection of cellularity, which seems to be more sensitive indicator of growth plate activity than its surface area.

BME signal is an unspecific MRI finding showing increased water content in the bone marrow accompanying fracture, stress reaction, bone contusion, inflammation, or tumour. Therefore the correlation with other imaging and clinical findings is necessary. The few areas of BME, found in three cases, most probably represent an unspecific stress reaction that was previously already described by Soder et al. in their study of asymptomatic adolescent soccer players [30]. Preserved integrity of menisci as well as the lack of larger joint effusion, cartilage, or cruciate lesion is in accordance with previous studies of MRI findings in asymptomatic junior athletes [31, 32].

The presented study has the following limitations. The study group was rather small therefore necessitating the confirmation of the results by a prospective study on a larger number of sporting and nonsporting adolescent subjects. To minimize bias resulting from consensus analyses by the MSK radiologist and the orthopaedic surgeon, MRI analyses were performed 2 months after the last MRI. Still, this might represent another study limitation. DWI with low *b* value (so-called “black blood” images) is susceptible to influences related to perfusion and T2. However we were cautious not to lose signal also in the growth plates with higher *b* values, being aware of biexponential behaviour of the signal intensity decay observed with increasing *b* values [15], in particular in the context of a limited experience in DWI of growth plates. Calculating the average ADC and growth plate area from several slices could be more accurate, but it would be also time-consuming and more complex for data analyses. The main advantage of DWI is in its quantitative nature that limits the observer's variability only to ROI delineation. In clinical routine, the articular cartilage, menisci, effusion, and cruciate ligaments are evaluated in both coronal and sagittal plane in one of standard sequences. We did not perform PD sequence in the sagittal plane for the time reasons; therefore some smaller lesions and effusions could have been missed. However, they were not clinically considered in any of the participants. To date, we have no data available on the kinetics and duration of ADC changes in the growth plates after an activity. To answer this question properly, a continuous daily scanning would be required. We currently also do not have any data on the normal values of ADC in growth plates of certain age groups; however, pre- and postactivity imaging allowed us to detect relative changes in ADC.

## 5. Conclusions

The presented study confirmed the feasibility of DWI in the evaluation of growth plates. Quantitative DWI measurements indicate increased cellularity in the medial part of the proximal tibial growth plate around the knee linked to intense soccer training in asymptomatic adolescent players. This suggests an asymmetric growth plate involvement that may consequently lead to bow-leg deformity. No detectable differences on a standard PD-FSE sequence were observed. Having a quantitative imaging tool for growth plates evaluation is important to delineate harmful sporting activities and to avoid or modify them accordingly to prevent long-term impacts on the growing skeleton.

## Figures and Tables

**Table 1:** General and anthropometric data of adolescent soccer players enrolled in the study (*N* = 11). Data are presented as mean (SD).

Data	Values (SD)
Height (cm)	169.2 (4.9)
Weight (kg)	59.3 (4.1)
BMI (kg/m^2^)	20.7 (1.3)
Lower limb alignment (mm)^*∗*^	23.6 (16.2)
Years of training	6 (1.3)
KOOS score SPORT	100 (0)
Tegner Lysholm Knee Score	100 (0)

^*∗*^Average intracondylar (positive for varus) or intramalleolar (negative for valgus) distance.

**Table 2:** Measured values of the central growth plate surface area (CGPSA) in mm^2^ and the calculated apparent diffusion coefficient (ADC) values in mm/s × 10^−3^ around the knees (*N* = 18) of adolescent soccer players before and after an intensive activity. Data is presented as mean (SD); statistically significant pairs are marked with *∗*.

Growth plate region	Central growth plate surface area		Apparent diffusion coefficient	
	Preactivity	Postactivity	Preactivity	Postactivity
Distal femur medial	265.28	261.17	1.3679	1.3473
Distal femur lateral	277.67	272.28	1.2749	1.2202
Proximal tibia medial	193.33	191.67	1.1333^*∗*^	1.0317^*∗*^
Proximal tibia lateral	213.72	210.39	1.2759	1.2177
